# Neural networks for computing and denoising the continuous nonlinear Fourier spectrum in focusing nonlinear Schrödinger equation

**DOI:** 10.1038/s41598-021-02252-9

**Published:** 2021-11-24

**Authors:** Egor V. Sedov, Pedro J. Freire, Vladimir V. Seredin, Vladyslav A. Kolbasin, Morteza Kamalian-Kopae, Igor S. Chekhovskoy, Sergei K. Turitsyn, Jaroslaw E. Prilepsky

**Affiliations:** 1 Aston Institute of Photonic Technologies, Aston University, Birmingham, B4 7ET UK; 2 Novosibirsk State University, Novosibirsk, Russia 630090; 3 National Technical University “Kharkiv Polytechnic Institute”, Kharkiv, 61102 Ukraine; 4 Federal Research Center for Information and Computational Technologies, Novosibirsk, Russia 630090

**Keywords:** Fibre optics and optical communications, Nonlinear optics

## Abstract

We combine the nonlinear Fourier transform (NFT) signal processing with machine learning methods for solving the direct spectral problem associated with the nonlinear Schrödinger equation. The latter is one of the core nonlinear science models emerging in a range of applications. Our focus is on the unexplored problem of computing the continuous nonlinear Fourier spectrum associated with decaying profiles, using a specially-structured deep neural network which we coined NFT-Net. The Bayesian optimisation is utilised to find the optimal neural network architecture. The benefits of using the NFT-Net as compared to the conventional numerical NFT methods becomes evident when we deal with noise-corrupted signals, where the neural networks-based processing results in effective noise suppression. This advantage becomes more pronounced when the noise level is sufficiently high, and we train the neural network on the noise-corrupted field profiles. The maximum restoration quality corresponds to the case where the signal-to-noise ratio of the training data coincides with that of the validation signals. Finally, we also demonstrate that the NFT b-coefficient important for optical communication applications can be recovered with high accuracy and denoised by the neural network with the same architecture.

## Introduction

Quite often, the evolution of nonlinear systems is well approximated by the nonlinear partial differential equations (PDE). Evidently, there is no universal theory for the solution of nonlinear PDEs, but there exists a distinguished class of nonlinear equations that can be solved with a mathematical rigour: the so-called *integrable systems*. The history of integrable PDEs started in the 1960s when Gardner et al.^1^ discovered a method for finding the infinite families of exact solutions for the Korteweg-de Vries equation. Their method termed the inverse scattering transform, can be deemed as the generalisation of the conventional Fourier transform (FT) to the nonlinear systems. Thus, the name nonlinear Fourier transform (NFT) for it is often used nowadays, especially in the signal processing literature^2,3^. Shortly after the integration of the Korteweg-de Vries equation, Zakharov and Shabat developed the inverse scattering machinery (i.e. the NFT method) for yet another celebrated PDE: the nonlinear Schrödinger equation (NLSE)^4^, which will be the focus of our current study.

In a nutshell, for an integrable PDE there exists the canonical transform of dependent variables, converting the original nonlinear system into the so-called action-angle variables; the evolution of the latter is governed by a set of uncoupled trivial (linear) differential equations. Mathematically, this can be treated as the effective linearisation of a nonlinear integrable PDE^5,6^. For our work, it is important that we know the explicit form of the NFT operations attributed to the NLSE.

The NLSE, being a generic model describing the interplay between the dispersive and nonlinear effects, is applicable to the description of a vast number of physical phenomena, ranging from the dynamics of magneto-ordered systems^7^ to hydrodynamics^8^. It also serves, under certain assumptions, as a principal master model governing the evolution of a single-polarisation slow-varying light envelope propagating along the single-mode fibre^9,10^. In the dimensionless form we write down the NLSE as:

1 $$\begin{aligned} i \frac{\partial q}{\partial z} + \frac{1}{2}\,\frac{\partial ^2 q}{\partial t^2} + |q|^2 q = 0 , \end{aligned}$$

In the fibre-optic context, *q*(*z*, *t*) is the electromagnetic field evolving down the fibre, *z* is the distance along with the fibre, while *t* is the retarded time variable. Eq. (1) is explicitly written as the focusing NLSE, corresponding to the anomalous dispersion of the standard optical fibre. We note that our further results are general and can be used for various physical applications, where NLSE (1) provides a good approximation. Nonetheless, without loss of generality, we will refer in the paper to the field *q* as to “a signal”.

Withing modern optical communications, the NFT is used not as a tool for the NLSE solution, but as a signal processing method^2,3^. This concept originated from the work of Hasegawa and Nyu^11^, who proposed to depart from considering the time domain solitonic shapes^10^, but rather use the nonlinear spectrum (the so-called eigenvalues) for the data modulation and transmission. Over the last decade, the NFT-based optical transmission techniques have been resurrected and greatly extended^3,12^. The most efficient NFT-based optical transmission method is the so-called nonlinear frequency division multiplexing (NFDM)^2^, within which we directly modulate the parameters of the nonlinear modes that emerge from the nonlinear Fourier (NF) signal decomposition. When the optical field propagates down the fibre link, the evolution of the nonlinear modes inside the NF domain stays almost linear, in contrast to the truly nonlinear evolution of signal in the space-time domain. Due to this property, we can theoretically get rid of the infamous nonlinear cross-talk degrading the transmission performance at high signal powers^13^.

**Figure 1:**
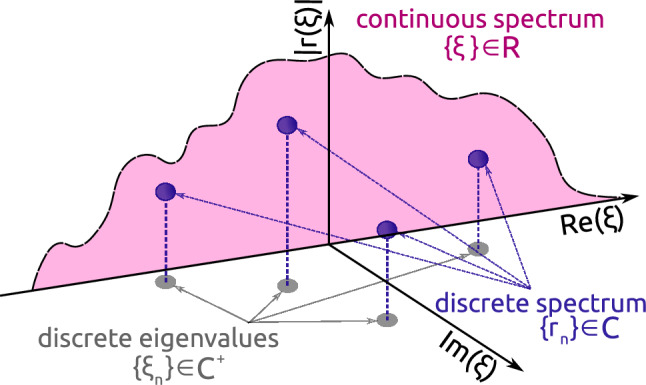
The schematic showing the different coexisting parts of a general NF spectrum: the discrete part, represented by the eigenvalues $\xi _n$ and respective norming constants $r_n$, and the continuous part, shown as the function $|r(\xi )|$ on the real $\xi$-axis.

Generally, when considering the NF decomposition of an arbitrary rapidly decaying wave-form, we can have two distinct coexisting parts of the NF spectrum: the continuous part, describing quasi-linear dispersive waves, and the discrete part, corresponding to solitonic modes^2,3,5,6^. The continuous part of NF spectrum is represented by the complex-valued function $r(\xi ) \in {\mathbb {C}}$ of a real argument $\xi \in {\mathbb {R}}$, where $\xi$ is called the spectral parameter; $r(\xi )$ is called the reflection coefficient, and $\xi$ emerges as the nonlinear analogue of a conventional Fourier frequency. This NF spectrum part converges to the conventional FT of our signal in the low-power limit^14^, see also the explicit expressions in Methods. The discrete part consists of the complex eigenvalues $\xi _n \in {\mathbb {C}}^{+}$, located in the upper complex half-plane, and the associated norming constants $r_n$ (spectral amplitudes)^15^. The graphical summary of the general NF spectrum structure is given in Fig. 1. However, we point out that it is exactly the utilisation of the *continuous NF spectrum part*^16–23^ that resulted in the breakthrough in the NFDM technology: this idea, mentioned already in early NFT transmission-related works^2,14^, is in stark contrast with the progenitor soliton-based transmission methods^10^. In our current study we specifically address the continuous NF spectrum: our goal is to compute the profile $r(\xi )$ given the localised *q*(*t*) shape. Then, we mention that the continuous NF spectrum modulation using the special technique coined b-modulation^24–27^ has provided the highest NFDM data rates so far^12,28^. Thus, in this paper we also address the recovery of the b-coefficient, $b(\xi ) \in {\mathbb {C}}$, $\xi \in {\mathbb {R}}$, given *q*(*t*). When the solitons are absent, as it is in the case considered, the full NF spectrum corresponding to a given finite-extent signal can be equivalently represented by either the reflection coefficient or by the b-coefficient, see more in Methods. Finally, we note that for the NFDM based on the discrete NF spectrum^29–31^, the achieved data rates have been noticeably lower than those for the modulation of continuous NF spectrum, see the comparison in^12^, Fig. 1], and we do not address the computation of solitonic parameters in our research.

The NFDM transmission method relies on the (approximate) integrability of our transmission channel, i.e. we inherently assume that Eq. (1) is a very accurate model describing the signal evolution down the fibre. However, aside from second-order dispersion and Kerr nonlinearity present in (1), in realistic fibre-optic systems, there are numerous other effects affecting the signal propagation. Optical noise inevitably arising during the amplification process^9^ is one of the key challenges in optical communications. The noise results in random NF spectrum disturbances^32,33^, imposing limits on the NFDM transmission quality. Thus, in our current work, we analyse the capability of a neural network (NN) to denoise the NF spectra. Similarly to Ref.^34^, in optical transmission applications, the NFT-Net that we consider in this work, is supposed to be integrated into the receiver architecture: it takes in the corrupted signal and yields the “purified” nonlinear spectrum containing the modulated data. Another widespread deviation from idealised model (1) is the non-zero nonuniform gain-loss profile occurring in realistic systems for both lumped^18,22^ and distributed^19^ amplification schemes. We also mention the effects of polarisation mode dispersion^35,36^, higher-order chromatic dispersion^35^, and component-induced impairments, to itemise just several important sources. All these effects bring about the deviations of the true optical channel from integrable NLSE (1) such that the NF spectrum of the signal at the end of our transmission system can be significantly distorted, which results in the appearance of errors in the transmitted data^20,21,35^. Given that, the machine learning and artificial neural networks (NN) based signal processing methods have recently attracted much attention, as they can effectively render adaptive distortions-resilient signal processing tools, and, thus, using the NNs we can mitigate the impact of detrimental factors mentioned above^37,38^.

The first direction in utilising the NNs for NFDM systems consists in applying the additional NN-based processing unit at the receiver to compensate the emerging line impairments and deviations from the ideal model^39–43^. But, despite ensuing transmission quality improvement, this type of NN usage brings about the additional complexity of the receiver. In the other approach, the NFT operation at the receiver is entirely replaced by the NN element. It has been shown that this approach, indeed, results in a considerable improvement of the NFT-based transmission system functioning^31,34,44^. But, despite the benefits rendered by such a NN utilisation, the NNs emulating the NFT operation have so far been mostly used in the NFDM systems operating with solitons only, and the NN structure used there was relatively simple. In the only work related to the continuous NF spectrum recovery^45^, a standard “imageInputLayer” NN (developed originally for hand-written digits recognition) from MATLAB 2019a deep learning toolbox was adapted to process the signals of a special form. Such an approach, evidently, has limited applicability and flexibility and is not optimal neither in terms of the result’s quality nor in the complexity of signal processing. In our current work, we demonstrate how this direction can be significantly extended and optimised, presenting and analysing the NN-based NFT modelling for the continuous NF spectrum, and using the special optimisation tools for finding the best NN architecture. We believe that our current research can lay the basis for the development of high-efficiency channel-agnostic NFDM transmission systems. Moreover, in our study, we address the question of recovering not only the NF spectrum $r(\xi )$, but also the b-coefficient, so it can be combined with the most efficient NFDM transmission method: the b-modulation.

Finally, we note that, recently, the interest in using the NFT as a signal-processing tool has risen in fields that are not directly relevant to optical transmission. In particular, the NFT was applied in the so-called integrable turbulence to monitor the appearance of coherent structures, such as breathers, solitons, and rogue waves^46,47^, to the optical microresonators regime analysis^48^, to the optical frequency combs characterisation^49^, and to the analysis of laser regimes and the emergence of dissipative coherent nonlinear structures^50–52^. The analysis of NFT modes’ evolution for such systems often appears to be more informative and convenient than dealing with the conventional Fourier modes. The NFT is also an important tool for the design of fibre Bragg gratings^53,54^. Thus, we believe that the technique presented in this work can have a much wider range of applications than simply being a processing tool in optical communications. To end up, solving nonlinear differential equations itself by using NNs is a fast-growing area with a range of applications in science and engineering^55–57^. We hope that our work will also advance knowledge in this emerging field.

## Results

In this section, we describe the main results obtained in the process of finding a suitable NN architecture for computing the continuous NF spectrum of a given signal. First, we describe which type of signals we used in training and testing. Next, we discuss the Bayesian optimisation application for our finding the best-performing NN architecture and the respective training procedure. Then, we analyse the output accuracy for the proposed NN architecture and compare it with that produced by a deterministic NFT numerical algorithm. In this paper, for the data generation and “conventional” computation we use the Fast NFT (FNFT) library^58^. At the end of the Results section, we show that the proposed NN architecture can predict not only the scattering coefficient $r(\xi )$, but also the NF coefficient $b(\xi )$, Eq. (8).

### Training data generation

In this work, without loss of generality, we analyse the NF decomposition of the signals having the form of wavelength division multiplexing (WDM) format with random modulation and return-to-zero carrier functions, considered in^59,60^. In the time domain, one (normalised) WDM symbol to decompose is given as the sum of independent subcarriers:

2 $$\begin{aligned} q(t)= & {} \frac{1}{Q} \sum _{k=1}^{M} C_k \, e^{i \omega _k t} f(t) {,} \quad -\frac{T}{2} \le t< \frac{T}{2} {,} \nonumber \\&\quad {{\text {with}}} \, \, \, f(t)= \left\{ \begin{array}{ll} \frac{1}{2}\Big [1 - \cos \left( \frac{4\pi t}{T} + 2\pi \right) \Big ] {,} &{}{{\text {for }}} -\frac{T}{2} \le t \le -\frac{T}{4} \; \; {{\text {or}}} \; \; \frac{T}{4} \le t \le \frac{T}{2}, \\ 1 {,} &{} {{\text {for }}} -\frac{T}{4}< t < \frac{T}{4}, \\ \end{array} \right. \end{aligned}$$

where *M* is a number of WDM channels, $\omega _k$ is a carrier frequency of the *k*-th channel, $C_k$ corresponds to the digital data in *k*-th channel, and *T* defines the symbol interval; *f*(*t*) is the carrier support waveform of our return-to-zero pulses. *Q* in (2) is the normalisation factor that we use to set the required energy for each signal (the total signal energy is calculated according to Eq. (3)). Each $C_k$ in (2) is a complex number drawn from the constellation with a particular cardinality, i.e. it is chosen with an equal probability from the finite set of allowed constellation points. For our NF decomposition analysis each time we use a single signal of the form given in Eq. (2). To train the NN, we precomputed 94035 such signals, with $C_k$ for each carrier randomly drawn from quadrature phase-shift keying (QPSK) constellations, i.e. the constellations with 4 possible points; the number of optical channels (carriers) in (2) is 15. Then we sampled our signal at equidistant points in time, $t_m$, over the segment of length *T*, $q(t_m)=q_m$: the number of sample points in each signal representation was $2^{10}= 1024$. The normalised symbol interval *T* was set to unity so that the time step size used was $\Delta t = 2^{-10}$ (for the explicit normalisations referring to single-mode fibre transmission see, e.g., Ref.^3^). For generated discretised profile, the reflection coefficient $r(\xi )$ was identified for 1024 sample points in $\xi$ variable, calculated using the fast numerical NFT method^58^. The parameter $\xi$ for our computations ranged from $-\pi / (4 \Delta t) \approx -804$ to $\pi / (4 \Delta t) \approx 804$: this region corresponds to the conventional Fourier spectrum computational bandwidth for the given sampling rate $\Delta t$, up to the scaling factor 2 referring to the linear limit correspondence^14^. Each signal in the dataset was eventually normalised so that its energy $E_{{{\text {signal}}}} = 39.0$. Some of the signals in the initial dataset for this energy contained solitons, but such signals were singled out and removed from the training and validation datasets. The remaining 94,035 signals did not contain solitons, which means that the discrete nonlinear spectrum for each signal is absent, such that these are used in our analysis. We note that although there are no solitons in the signals, we are still operating in the regime where the signal nonlinearity is not negligible, see Methods. The more straightforward way of generating the datasets with desired properties would be to use the inverse NFT routines, but these are much more time-consuming, such that we decided to employ the data-generation approach described below: it also allows us to explicitly control the accuracy of the generation process.

Together with the set of deterministic signals, we generated the signal sets with the addition of uncorrelated Gaussian noise, adding the random value to each sample point. In realistic applications, the source of this noise can be the instrumental imperfections of the transceiver or the effects relevant to inline amplifiecation^9^. The signal-to-noise ratio (SNR) is a traditionally used characteristic for quantifying the level of a noisy corruption:

3 $$\begin{aligned} {{\text {SNR}}} = \frac{E_{{{\text {signal}}}}}{E_{{{\text {noise}}}}} {,} \qquad E_{{{\text {signal}}}} = \sum _{m = 0}^{N - 1} |q_m|^2 \Delta t {,} \end{aligned}$$

where $E_{{{\text {signal}}}}$ and $E_{{{\text {noise}}}}$ are the signal and noise energies, respectively; $q_m$ is the *m*-th signal sample, with *N* being total number of sampling points, $\Delta t$ is the time sample size. For further training, in addition to the set without noise, which had 84632 signals, we used 8 sets of 423160 signals (5 different noise realisations). Each set corresponds to one of the following SNR values: $\{0, \, 5, \,10, \, 13, \, 17, \, 20, \, 25, \, 30\}$ dB. 9 sets of 9403 signals with the corresponding noise levels were left to validate the network performance. Validation data sets were not used in the training process. We note that the NFT in optical communications is tailored for use in long-haul systems, meaning the high levels of noise (low SNR) is most interesting from the application perspectives. However, we also include the results for high SNR levels to analyse the NN functioning peculiarities in detail.

### Neural network design and Bayesian optimisation

As mentioned above, the general NF spectrum attributed to a given localised waveform consists of two parts: the discrete spectrum that we do not consider in our current study (our trial pulses do not contain any solitonic component, neither in pure form nor in the noisy case), and the continuous part which is our subject in hand here. The continuous part is retrieved through considering the special Jost solutions (7) to the Zakharov-Shabat problem (6), see Methods. The goal of our work is to demonstrate the fundamental possibility of replacing the direct calculation of NF spectrum through the numerical solution of the Zakharov-Shabat problem (6) with the computations employing specially-designed and trained NNs.

The latter task can be addressed using the encoder-decoder approach, where the encoder transforms the input signal into some intermediate vector representation and, later, the decoder converts this representation into the output signal. We notice that the input and output signals can belong to two different data domains. There are several advantages of this approach, e.g. it is quite flexible, so the encoder and decoder structures can differ to match exactly the “nature” of each signal’s domain. With this, we train such NNs in the end-to-end style, so the weights of the encoder and decoder will be trained simultaneously and fit each other. A lot of highly efficient encoder-decoder architectures have been designed up to date, e.g. those can demonstrate an efficiency higher than that of a human brain for some specific tasks^61^. For processing quite long sequences (typically more than 1000 data points), the convolutional NNs (CNN) are often more beneficial than the recurrent NNs (RNN). Also, the CNN allows us to parallelise the computations in an efficient way, which is important in our case. Thus, we argue that the encoder-decoder architectures based on CNNs are most suitable for our data and task, though other NN types may also deserve investigation in latter studies.

As a starting point, we took the WaveNet^62^-based network, which extends the concept of deep CNNs. Models of this type have several advantages, among which we underline the reduction of time required for training the network on long data sequences. However, a significant drawback of this architecture is the requirement to embed a large number of convolutional layers to increase the receptive field. In our work, to increase the effective size of that region, we used convolutions with dilation. This made it possible to exponentially increase the receptive field with the NN depth growth and, therefore, to capture a larger number of data points in the input signal.

The momentous issue in using NNs to perform any nonlinear transformation is the choice of the optimal network architecture. One of the optimisation methods is to enumerate the possible combinations of NN parameters. But even in the case of a relatively small number of layers, the number of hyperparameters can reach several thousand, which makes the optimisation process very time-consuming, if realisable at all. Thus, the search for an optimisation algorithm for such computationally expensive problems can be extremely difficult. However, the Bayesian optimisation method^63^ is deemed to be one of the most efficient optimisation strategies, and so we employ it in this work to find the optimal hyperparameters distribution for the NFT-Net.

**Figure 2:**
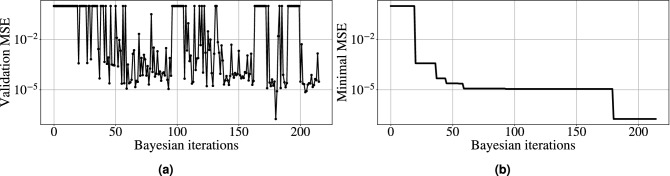
**(a)** The dependence of the mean squared error (MSE) value on the number of Bayesian iteration. **(b)** The same for minimal value of MSE.

The Bayesian optimization builds a probabilistic model of the function mapping from hyperparameter values to the objective evaluated on a validation set^63,64^. By iteratively evaluating a promising hyperparameter configuration based on the current model, and then updating it, the Bayesian optimization aims to gather observations revealing as much information as possible about this function and, in particular, the location of the optimum. Thus, it tries to balance exploration (hyperparameters for which the outcome is most uncertain) and exploitation (the hyperparameters expected to bring us close to the optimum). An important aspect to note is that the Bayesian optimisation often does not return one specific point in the parameter hyperspace for which the optimised function is minimal. The process converges into some subspace of parameters, where several points can locally minimize the function^63^. A detailed description of hyperparameters tuning can be found in the article^65^ where Bayesian optimisation is used to adapt parameters for the synthesis of a digital pre-distortion filter for optical transmitters.

**Figure 3:**
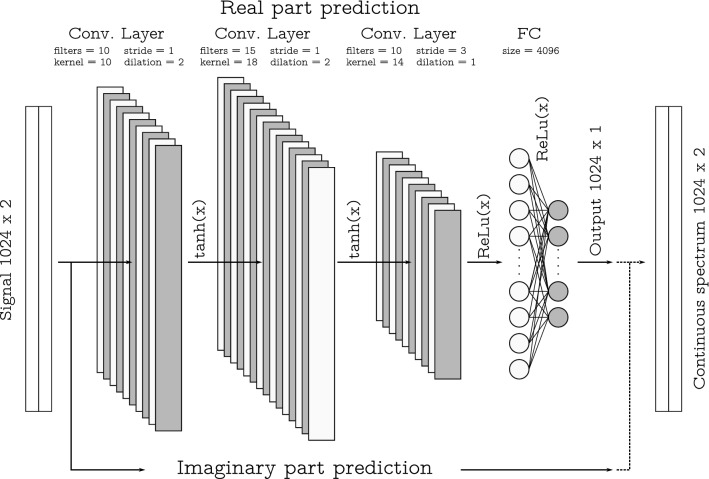
The schematic of NFT-Net topology: the extended scheme presents the sequence of operations for the processing of real part; the processing of imaginary part is identical (marked with the long arrow below the scheme). The numbers indicating the layers/arrays sizes refer to our processing 1024 complex-valued signal samples.

We manipulate the following hyperparameters for the convolutional part of the neural network: the number of convolutional layers, the number of filters, the kernel size, stride, dilation, and the activation function for each layer. We used the activation functions “ReLU”, “tanh” and “sigmoid” in the hyperparameters optimisation. After the convolutional part, there are 2 fully connected layers, the second of which has a fixed size (1024, which corresponds to the size of the output vector). The size and activation function of the first fully connected layer was also a hyperparameter for optimization. For the optimisation, we used a dataset without additional noise and employed only the real part of the continuous spectrum for the prediction. After that, the “optimal” architecture (but not weights) is fixed, and is no longer changed to predict the imaginary part of the continuous spectrum or for our operating with the datasets with additional noise. The loss function was optimised for each architecture. We used the mean squared error (MSE) as the loss function, aiming to minimise the MSE between the network output and the target output computed with the conventional NFT method^58^. In training, we employed the Adam (Adaptive Moment Estimation) optimisation algorithm with the learning rate of 1e–4^66^. The learning process of each point in the parameter hyperspace was stopped if the value of the loss function did not decrease over 5000 epochs. We chose this large epoch stopping-criterium number to neutralise the factor of randomness in the learning process, which appears due to the random choice of the initial weights. Additionally, we checked the value of the loss function on the validation set to prevent the overfitting, but for the amount of training data used, the overfitting was not observed. Figure 2 presents the dependence of the MSE value and dependence of its minimum on the Bayesian iteration number. For architectures with more than 20 million training parameters, we set the value of the loss function to 1.0: this explains the upper cut-off limit in the figure. It is apparent from Fig. 2 that the optimisation has identified a subspace where many architectures have approximately the same value of the loss function at the level of $10^{-5}$. However, there was a point where the value was at the level of $10^{-7}$. Thus, we took this point (a set of hyperparameters) as the optimal one. After finding the optimal architecture, each NN’s weights were trained for different SNR but keeping the same optimal architecture parameters. On average, with the amount of data used, our learning process took 50,000 epochs to reach the minimum for each noise level.

**Figure 4:**
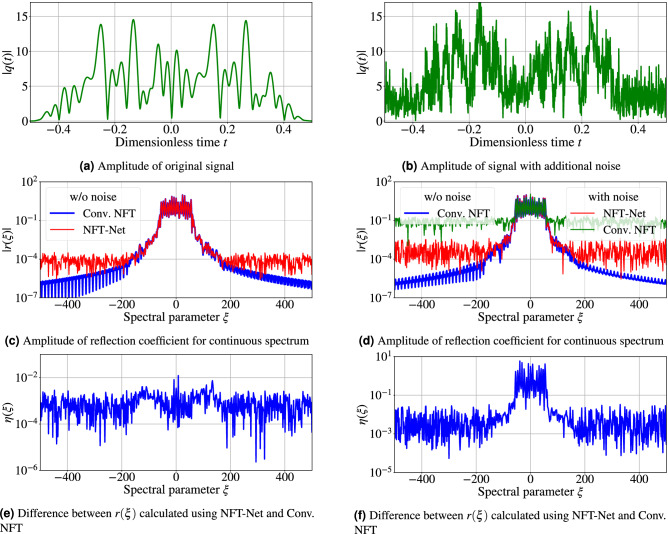
Panel **(a)** shows an exemplary amplitude of a original complex WDM signal *q*(*t*) versus time. Below (panel **(c)**) is the amplitude for calculated scattering coefficient $r(\xi )$ associated with the signal from the pane above. The blue line corresponds to the data obtained using the conventional NFT method, the red line corresponds to the NFT-Net result. The difference between the scattering coefficients for signal example calculated by these methods is shown in panel **(e)**. Pane **(b)**: the same plot for complex signal *q*(*t*) with the addition of Gaussian noise. The SNR value used is 5 dB. Plot **(d)** shows the result of calculating the continuous spectrum for the noisy signal using the FNFT method (green line) and using the NFT-Net trained at the same noise level (SNR = 5 dB, red line) and original spectrum (for noiseless case, blue line). For NFT-Net trained with noise, pane **(f)** below shows the difference between the predicted scattering data for the example of noisy signal and the reflection coefficient calculated by conventional NFT for that signal without noise.

The original signal and NF data for the continuous spectrum are complex-valued functions. Therefore, two networks with the same architecture are to be used for the whole transformation; each identical part is responsible for the computation of either the real or imaginary parts of the resulting arrays, which contain the values of continuous NF spectrum defined in Eq. (10). Figure 3 depicts the schematic for the entire optimised NFT-Net architecture. The convolutional part consists of three layers with 10, 15 and 10 filters. Kernel sizes of the first and third convolutional layers are 10, and for the layer between them, it is 18. As noted above, we took the dilation value for each layer as one of the sought hyperparameters. For NFT-Net, the optimisation gave that the first two layers have dilation 2, stride 1 and “tanh” activation function, and for the third layer, the dilation is 1 with stride 3 and “ReLu” activation. After the CNN part we put the flattening layer, not shown in the figure (but affecting the processing complexity), and two fully-connected layers with 4096 and 1024 neurons. The exemplary picture of how the designed NN works on one signal is given in Fig. 4c. In this figure, we show the results of the NN-based NF spectrum computation for the noiseless case. Already from this figure, we can notice that the result produced by our NN and that obtained from conventional NFT routine^58^ are very similar.

### Studying the NFT-Net performance for computing NF spectra of noisy signals

In this section we analyse the NFT-Net performance and the denoising property of the NN. We compare the deviations in the obtained nonlinear spectrum calculated with the NFT-Net and calculated with the conventional NFT applied to the same signal without noise. To quantify the performance rendered by the NFT-Net application with the performance of conventional algorithms applied to noisy signals, we use the following metric:

4 $$\begin{aligned} \eta = \frac{1}{S} \, \sum _{i = 1}^{S} \langle \eta _i(\xi ) \rangle _{\xi }, \quad \eta _i(\xi ) = \frac{|\{r_{{\text {predicted}}}(\xi )\}_i - \{r_{{\text {actual}}}(\xi )\}_i| }{\langle |\{r_{{\text {actual}}}(\xi )\}_i| \rangle _{\xi }} {,} \end{aligned}$$

where *S* is the total number of signals in the validation set, $\langle \cdot \rangle _{\xi }$ denotes the mean over the spectral interval, $\{r_{{{\text {predicted}}}}(\xi )\}_{i}$ and $\{r_{{{\text {actual}}}}(\xi )\}_{i}$ correspond to the value of reflection coefficient $r(\xi )$ computed for the signal number *i* at point $\xi$ (we compare the quantities for the validation data set). The label “predicted” refers to the result produced by the NFT-Net on the noisy signal, and “actual” marks the $r(\xi )$ value obtained using the conventional NFT algorithm^58^ for the noiseless signal. The relative error $\eta (\xi )$ is determined at the point $\xi$, so we use $\langle \eta (\xi ) \rangle _{\xi }$ to estimate the overall mean of the error for one signal, and use Eq. (4) to evaluate the error for the entire validation dataset. We stress that the metric was chosen in such a way as to take into account even the regions where the value of the spectrum is much less than one.

The results of our comparison for $r(\xi )$ computation using different SNR levels for NFT-Net are presented in Table 1, and are arranged as follows. The first column of the table identifies the SNR value in dB for the validation signals, i.e. the level of noise for the signals which we analyse. The first row of the table displays the SNR values of noisy signals from the training set, i.e. it shows the noise level of the signals on which the NFT-Net was trained. We notice that the case ${{\text {SNR}}}=30$ dB corresponds to almost negligible noise, while for ${{\text {SNR}}}=0$ dB our noise energy is equal to that of our signal, which signifies a very intensive noisy corruption. Thus, each column in the table corresponds to the results produced by the NN trained on the signals with the chosen level of a noisy corruption. The number in each cell shows the averaged metric value, Eq. (4), where for the computation of $\{r_{{{\text {predicted}}}}(\xi )\}_{i}$ we used the NFT-Net trained on the signals with SNR values shown in the first row and applied to the validation signals having the SNR values given in the first column. The “Conv. NFT” column shows the error value for the numerical result of the fast NFT method on the signals with added noise, where the respective SNR is presented, again, in the first column. The value of metric (4) corresponding to the conventional NFT method applied to noiseless signals is, obviously, zero: the results provided by the conventional NFT without noise are taken as the true ones. When the NFT-Net produces a less accurate result compared to the conventional NFT applied to the noisy signal, the cell is marked with bold; when the NFT-Net outperforms the conventional NFT method, i.e. it successfully purifies our signal from noise, the respective cell is not highlighted (white). Whence, the white area size in each table demonstrates how well the NFT-Net retrieves the nonlinear spectrum for noise-corrupted signals.

**Table 1:** Comparison of the NFT-Net performance against the conventional NFT in the computation of coefficient $r(\xi )$.

		Conv. NFT	Training SNR level, dB								
			w/o noise	30	25	20	17	13	10	5	0
Validation SNR level, dB	w/o noise	0	**8.39e–4**	**6.52e–3**	**9.43e–3**	**1.26e–2**	**1.61e–2**	**2.38e–2**	**3.59e–2**	**7.43e–2**	**1.42e–1**
	30	6.91e–2	5.54e–2	9.56e–3	1.11e–2	1.36e–2	1.68e–2	2.42e–2	3.63e–2	**7.49e–2**	**1.44e–1**
	25	1.23e–1	9.84e–2	1.40e–2	1.39e–2	1.51e–2	1.78e–2	2.45e–2	3.63e–2	7.47e–2	**1.43e–1**
	20	2.21e–1	1.74e–1	2.53e–2	2.18e–2	1.97e–2	2.08e–2	2.58e–2	3.65e–2	7.40e–2	1.43e–1
	17	3.10e–1	2.41e–1	3.96e–2	3.23e–2	2.63e–2	2.53e–2	2.78e–2	3.70e–2	7.31e–2	1.42e–1
	13	4.89e–1	3.66e–1	7.74e–2	6.12e–2	4.53e–2	3.97e–2	3.54e–2	3.98e–2	7.06e–2	1.38e–1
	10	6.78e–1	4.88e–1	1.29e–1	1.03e–1	7.36e–2	6.23e–2	5.12e–2	4.85e–2	6.87e–2	1.33e–1
	5	1.16e+0	7.26e–1	2.73e–1	2.31e–1	1.72e–1	1.43e–1	1.15e–1	9.93e–2	7.98e–2	1.17e–1
	0	2.00e+0	9.48e–1	4.79e–1	4.37e–1	3.60e–1	3.12e–1	2.59e–1	2.29e–1	1.74e–1	1.16e–1

The table presents the results for the optimised NFT-Net architecture from Fig. 3. The values in the cells show the error value (4) for each specific pair of training and validation sets SNR. The bold cells correspond to the cases when the accuracy of the NFT-Net nonlinear spectrum restoration is lower than that of fast NFT, i.e. the NN does not denoise the signal well, while the white cells correspond to the cases when the accuracy of the continuous NF spectrum rendered by the NFT-Net is higher, i.e. the NN effectively denoises the result.

Table 1 shows the error values for the restoration of $r(\xi )$ coefficient (10) of a noiseless and noisy-perturbed signals (2), by the NFT-Net architecture given in Fig. 3. The first row in the table corresponds to the noiseless case. It is always marked with bold, which means that the NN cannot provide any better results than the benchmark ones rendered by the conventional fast NFT method used to generate the training data.

However, the values of the error for noise-corrupted signals reveal interesting tendencies. It follows from the table that for the low training noise level (up to 10 dB, columns three through nine), the NFT-Net error is typically lowest for the noiseless validation dataset (second row). Thus, the addition of low noise in the training dataset only degrades the NFT-Net restoration capability, even though this decrease is not significant. This NFT-Net feature can be deemed as the NN’s being “confused” by the weak noise in its training in the nonlinear transformation identification. For the most interesting case of high noise, the network works best for samples where the SNR value is the same for the validation and training sets. In such cases, the relative error is about 8–12%, while the error for conventional NFT is at the level of 100–200%. Another fact is that with decreasing noise (rows from bottom to top) in the validation set, the error value remains at approximately the same level after the cell corresponding to the same training and validation noise values. These results confirm that the presented NN architecture is capable of performing the desired nonlinear transformation, the NFT, and, in addition, it can also work as an effective denoising element when the noise level becomes non-negligible.

The examples of original and noise-corrupted signals and the corresponding nonlinear continuous spectra are given in Fig. 4, where we used the NFT-Net for the computations. Figures 4b and d show that when the additional noise distorts the signal, the conventional numerical algorithms naturally produce the noise-distorted nonlinear spectra. Fig. 4e and f show the relative error value $\eta (\xi )$ (4) for the continuous spectrum prediction with NFT-Net for the signal without noise (left) and the signal with noise (right), and the reflection coefficient computed for the original signal by the conventional NFT (marked as “Conv. NFT” in the panes’ legends). In Fig. 4c, e, the NFT-Net is trained on the dataset without adding noise, and in Fig. 4d, f, the NFT-Net is trained on the dataset with additional noise for SNR = 5 dB. Figure 4c and d show that in the presence of noise, the fast NFT results begin to deviate noticeably from the original (noiseless) values, while the NFT-Net tends to denoise the resulting nonlinear spectrum.

### NFT-Net performance for the restoration of NF coefficient $b(\xi )$ attributed to noisy signals

In addition to the coefficient *r*, from the optical communications perspective it is instructive and important to check how the proposed architecture would work to predict the NF coefficient *b*, Eq. (8). We note that the optical transmission method coined b-modulation^24,26,49^, where we operate with the modulation of the b-coefficient, has proven to be the most efficacious technique among different NFDM methods proposed^12,28^. Moreover, for the practical case when our signal has a finite extent, the continuous part of the NF spectrum can be completely described by the b-coefficient only, because the second NF coefficient $a(\xi )$ can be calculated from $b(\xi )$ profile, see Eq. (11) in Methods. Our goal here is to demonstrate that the same NFT-Net structures can be used for the both $r(\xi )$ and $b(\xi )$ computation, when the NN is trained on the respective dataset. As the loss function, we now use the MSE build on the b-coefficient samples, and the MSE is also used as our quality metric in the respective tables:

5 $$\begin{aligned} \eta _b = \frac{1}{S} \, \sum _{i = 1}^{S} \langle \eta _{b,i}(\xi ) \rangle _{\xi }, \qquad \eta _{b,i}(\xi ) = \frac{|\{b_{{\text {predicted}}}(\xi )\}_i - \{b_{{\text {actual}}}(\xi )\}_i| }{\langle |\{b_{{\text {actual}}}(\xi )\}_i| \rangle _{\xi }}. \end{aligned}$$

The notations are the same as we used in (4): the labels “predicted” and “actual” correspond, respectively, to the result of the NFT-Net applied to noisy signals and the result produced by the conventional NFT routine applied to noiseless signals.

We carried out the analysis of the NFT-Net performance for the restoration of b-coefficient using the same approach as we did in the previous subsection for $r(\xi )$. Our results for noise pulses with the different level of noise are summarised in Table 2. We checked that the NFT-Net configurations when applied to the computation and denoising of $b(\xi )$ revealed the same tendencies for the quality of restoration as we observed in the previous subsection devoted to the reflection coefficient $r(\xi )$.

**Table 2:** Comparison of the NFT-Net performance against the fast conventional NFT in the computation of coefficient $b(\xi )$.

		Conv. NFT	Training SNR level, dB								
			w/o noise	30	25	20	17	13	10	5	0
Validation SNR level, dB	w/o noise	0	**7.12e–3**	**5.37e–3**	**5.87e–3**	**6.49e–3**	**8.69e–3**	**1.07e–2**	**1.20e–2**	**1.58e–2**	**1.77e–2**
	30	1.15e–1	5.83e–2	6.73e–3	6.69e–3	7.16e–3	8.95e–3	1.08e–2	1.20e–2	1.58e–2	1.77e–2
	25	2.05e–1	1.02e–1	1.02e–2	8.70e–3	8.48e–3	9.54e–3	1.09e–2	1.21e–2	1.58e–2	1.77e–2
	20	3.64e–1	1.75e–1	2.13e–2	1.51e–2	1.18e–2	1.12e–2	1.14e–2	1.23e–2	1.59e–2	1.78e–2
	17	5.14e–1	2.38e–1	3.66e–2	2.41e–2	1.59e–2	1.33e–2	1.21e–2	1.26e–2	1.60e–2	1.78e–2
	13	8.14e–1	3.44e–1	7.73e–2	4.94e–2	2.64e–2	1.88e–2	1.39e–2	1.36e–2	1.63e–2	1.80e–2
	10	1.15e+0	4.41e–1	1.31e–1	8.58e–2	4.21e–2	2.70e–2	1.67e–2	1.51e–2	1.69e–2	1.84e–2
	5	2.04e+0	6.22e–1	2.70e–1	1.96e–1	9.95e–2	5.91e–2	2.81e–2	2.18e–2	1.95e–2	1.99e–2
	0	3.60e+0	7.98e–1	4.54e–1	3.68e–1	2.24e–1	1.42e–1	6.34e–2	4.43e–2	2.93e–2	2.43e–2

The table presents the results for the NFT-Net architecture from Fig. 3. The values in the cells show the error value (5) for each specific pair of training and validation sets SNR. The bold cells correspond to the cases when the accuracy of the NFT-Net nonlinear spectrum restoration is lower than that of fast NFT, i.e. the NN does not denoise the signal well, while the white cells correspond to the cases when the accuracy of the continuous NF spectrum rendered by the NFT-Net is higher, i.e. the NN effectively denoises the signal.

A similar situation as was observed for coefficient $r(\xi )$, remains in this case. The error is minimal for a noiseless validation set. However, this trend now continues for high noise levels. A similar tendency is observed all over the results: the values above the diagonal vary slightly. The additional observations when dealing the b-coefficient are as follows. An interesting difference from the case relevant to $r(\xi )$, is that the metric value (5) in the case of predicting $b(\xi )$ is less, and the bold region in Table 1 is larger compared to what we see in Table 2 for the b-coefficient. From the results, it is clear that the prediction accuracy is higher for the b-coefficient. It means that our NN generally works more accurately for the restoration of coefficient $b(\xi )$ than for $r(\xi )$. This result can be expected, as the noise-perturbed $r(\xi )$ contains the noisy contributions from both $a(\xi )$ and $b(\xi )$, while the b-coefficient involves only its noisy contribution, and thus gets corrupted less. So in the latter case, the NN has to clean off less noise.

**Figure 5:**
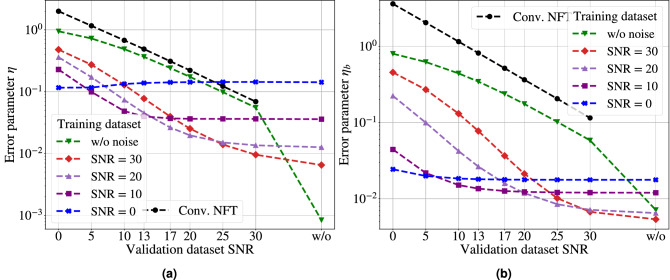
**(a)** The dependence of the error parameter $\eta$ (4) for coefficient $r(\xi )$ on the SNR value of the validation dataset for fast conventional NFT and NFT-Net trained at different noise levels. **(b)** The same for the error parameter $\eta _b$ (5) for coefficient $b(\xi )$. The black line represents the error value for fast NFT applied to noisy signals, and the points below this line refer to the cases when the NFT-Net outperforms conventional computations. Other lines show the value of the error in calculating the continuous spectrum using NFT-Net, trained with different noise levels: green—without additional noise, red—with additional noise with SNR = 30 dB, violet—at SNR = 20 dB purple—at SNR = 10 dB, blue—at SNR = 0 dB.

Figure 5 summarizes the above and shows the calculation errors (4) and (5) for NFT-Net architecture. The plot actually visualises the values and tendencies from Tables 1 and 2. For both $r(\xi )$ and $b(\xi )$ coefficients, the NN outperforms the fast NFT results when the NFT-Net gets trained on the data with additional noise.

## Discussion

Our goal in this work was to demonstrate that the NN can be successfully used for performing the NFT operation, in particular, for computing the profile of continuous NF spectrum. Note that our interest was not only the computation of the continuous NF spectrum, i.e. the nonlinear transformation, but the possibility to denoise signal using NNs. We started with the WafeNet-type architecture^62^, which is effectively a deep CNN, and applied Bayesian optimization^67^ to find the optimal set of hyperparameters. Initially, we set the task of optimizing the entire architecture, so the hyperparameters were not only the parameters of the layers but also their number.

Once again, we emphasize that Bayesian optimization does not always give the “best” set of parameters. It provides a subspace of hyperparameters in which neural networks with such parameters are best trained on the available dataset. Due to the fact that neural networks are universal approximators, any sufficiently complex architecture can be trained for a specific task. We can expect that the optimization process can converge endlessly towards increasing the complexity of the network. However, this is not suitable for our task, where we want to minimize the complexity of the network while improving the accuracy of the work. Therefore, we simultaneously limited the number of trainable parameters in the NN during optimization. In our case, during the optimization process, we found an architecture that gives us the best metric value (4) and we chose it as the desired architecture. Further, the optimization process could converge to another subspace of hyperparameters, but we stick to the point with the minimum value of the loss function.

We found that this NN, indeed, can perform the NFT operation and denoise the received NF spectrum: the denoising effect is pronounced at medium to high noise levels. To achieve this effect, several realisations of the noise are needed for the neural network to “understand” the influence of noise on the signal. As expected, denoising is typically best when the training and testing data noise levels coincide, though we observed some deviations from this rule for lower noise levels, where the quality of restoration of the NF spectrum also makes a noticeable contribution in the overall error value. When being trained on different noise levels, the NFT-Net was still able to produce denoising, thus demonstrating the design’s flexibility. We have shown that conventional NFT calculation methods give “distorted” results when working added noise. In fact, the “distorted” results are actually correct, but from the nonlinear transformation point of view. But from the application’s perspective, we are almost always interested in the denoised signals to reduce the embedded data corruption level. At this place we notice that the exemplary signals that we used for the NFT-Net training/testing, Eq. (2), are, evidently, different from those used in r- of b-modulated NFDM systems. Moreover, the latter are subject to dispersive effects as the NN has to process them at the receiver side after their having passed some distance. To adapt the NFT-Net for the different signals, two possible strategies can be used. The first one is straightforward, where we retrain the NN from scratch using a different dataset. The second strategy can make use of the pretrained NFT-Net model and utilise domain randomisation and adaptation^68,69^. We believe that after the retraining procedure, the NFT-Net (or some of its modifications, if we find that the capacity of the proposed NN architecture is insufficient to account for some complicated real-world effects) should be capable to account for the spurious soliton emergence and involved noise properties taking place in the realistic optical transmission systems.

Finally, we note that the problem of recovering a few solitons from a given pulse utilizing NN has been studied in^31,34,44,70^. However, the NN architectures used in those studies are much simpler as one has to identify and filter only a few solitonic parameters , while in our work we recovered 1024 complex numbers representing the continuous NF spectrum. A larger number of solitary modes was considered in^71^, where, however, only the total number of solitons in the pulse was studied. Potentially, it is interesting to combine the NN developed in our work with the additional module that can deal with soliton parameters restoration: such a hybrid tool would be able to perform the complete NFT decomposition of an arbitrary decaying pulse.

To sum up, we investigated the modelling of the NFT operation associated with the focusing NLSE, using the NN with a special structure, which we coined the NFT-Net. We considered here an almost unexplored case dealing with the computation of the continuous part of the NF spectrum. It was demonstrated that the WaveNet-type NFT-Net structure can satisfactorily perform the task of the NF spectrum computation, and the best-performing architecture was obtained by Bayesian hyperparameters optimisation. Moreover, we showed that the same NFT-Net structure can be used to efficaciously retrieve both the reflection coefficient $r(\xi )$ and the scattering coefficient $b(\xi )$. The most practically important feature of the developed NN-based method is its capability to perform signal denoising. We demonstrated that the NN-based processing can bring about essential improvements in the quality of NF spectrum restoration attributed to noise-perturbed time-domain profiles, compared to the conventional high-accuracy NFT processing method. The advantage in denoising becomes most pronounced at high noise levels, with the maximum restoration quality typically occurring when the SNR of the training data is the same as that of the validation dataset.

## Methods

### Forward NFT operation for focusing NLSE

The NF spectrum associated to a given pulse *q*(*t*) (we drop the dependence of our quantities on *z* for simplicity) having a finite $L_1$ norm, is calculated using the solutions of the so-called Zakharov-Shabat spectral problem^2–4,6^. The latter is represented by the set of coupled ordinary differential equations written for two auxiliary functions $v_{1,2}$. Our signal to decompose, *q*(*t*), enters into this set as an effective potential. We write down the Zakharov-Shabat problem (the focusing NLSE case) as^4^:

6 $$\begin{aligned} \frac{d}{dt} \left( \begin{matrix}v_1(t, \xi )\\ v_2(t, \xi )\end{matrix}\right) =\left( \begin{matrix}-i\xi &{}q(t)\\ -{\bar{q}}(t)&{}i\xi \end{matrix}\right) \left( \begin{matrix}v_1(t, \xi )\\ v_2(t, \xi )\end{matrix}\right) . \end{aligned}$$

In Eq. (6), $\xi$ is the (generally complex-valued) spectral parameter which plays the role of conventional Fourier frequency for integrable nonlinear PDEs. The overbar in Eq. (6) and below denotes the complex conjugates of corresponding quantities. To determine the NF spectrum associated with our profile *q*(*t*), we need to find the special solution $\Phi (t,\xi )$ of Eq. (6), called Jost function, imposing the special asymptotic condition at the trailing end of the pulse:

7 $$\begin{aligned} \Phi (t,\xi )\equiv \left( \begin{matrix}\phi _1\\ \phi _2\end{matrix}\right) \xrightarrow [t\rightarrow -\infty ]{} \left( \begin{matrix}e^{-i\xi t}\\ 0\end{matrix}\right) . \end{aligned}$$

The NF pulse decomposition consists in finding the continuous and discrete components of the NF spectrum associated with the localised signal *q*(*t*). The core part of NFT is the calculation of scattering coefficients, $a(\xi ) \in {\mathbb {C}}$ and $b(\xi ) \in {\mathbb {C}}$, defined through the Jost solution $\Phi (t,\xi )$ as follows

8 $$\begin{aligned} a(\xi )=\lim _{t\rightarrow +\infty }\phi _1(t,\xi ) e^{i\xi t}, \qquad b(\xi )=\lim _{t \rightarrow +\infty }\phi _2(t,\xi ) e^{-i\xi t}, \end{aligned}$$

where $\xi \in {\mathbb {R}}$. The scattering coefficients for the focusing NLSE satisfy:

9 $$\begin{aligned} |a(\xi )|^2 + |b(\xi )|^2 \equiv 1. \end{aligned}$$

The continuous part of NF spectrum is generally defined by the ratio of quantities *b* and *a* from (7):

10 $$\begin{aligned} r(\xi )=b(\xi )/a(\xi ), \qquad r(\xi ) \in {\mathbb {C}}, \end{aligned}$$

where $r(\xi )$ is often refereed to as the reflection coefficient. $r(\xi )$ plays the role of the ordinary Fourier spectrum for nonlinear integrable PDEs and converges to the FT of our signal in the low-power (linear) limit; see more direct expressions below.

The discrete part of NF spectrum (the solitonic degrees of freedom) consists of the set of complex-valued pairs: $\{ \xi _n, c_n\}$, where *n* numerates the soliton mode, and each $\xi _n$ is the (non-degenerate) solution of the equation $a(\xi )=0$, laying the the upper complex semi-plane of $\xi$. The second quantity, the so-called norming constants $c_n$, are given (for a sufficiently localised signal^72^) by: $c_n = c(\xi _n) = b(\xi _n)/a'(\xi _n)$, with prime meaning the derivative with respect to $\xi$. The value of $\xi _n$ determines the amplitude and frequency of each solitonic component, while $c_n$ defines the values of phase and the “centre-of-mass” position of a solitary mode. However, the discrete part of NF spectrum is not addressed in our study; see Refs.^31,34,44^ where the solitonic parameters are computed using the NNs.

More exact mathematical details regarding the NF spectrum definition and properties can be found in, e.g., monograph^6^, see also Ref.^72^ for a brief mathematical review.

### NF spectrum associated with finite-extent signals

In practical applications, we do not typically deal with the signals defined on the whole infinite *t*-axis, but rather operate with the truncated wave-forms, meaning that *q*(*t*) is non-zero only inside the finite interval of *t*. In this case, the NF spectrum of the signal is completely characterised by the coefficient $b(\xi )$ from (8), which becomes band-limited, appended with the finite discrete set of solitonic parameters $\{\xi _n,c_n\}$^25,26^. When, in addition, the discrete NF spectrum is absent, as it is in the case considered, the whole NF spectrum can be defined using just $b(\xi )$ profile^24^, while the coefficient $a(\xi )$ can be expressed through $b(\xi )$ in the following way:

11 $$\begin{aligned} a(\xi ) = \sqrt{1-|b(\xi )|^2} \, \exp \left[ \frac{i}{2 \pi } \intop ^{\infty }_{-\infty } \frac{\ln \big (1-|b(s)|^2 \big )}{\xi - s} \, ds \right] , \end{aligned}$$

where the integral in the exponent is understood in the principal value sense. So, in practice, instead of $r(\xi )$ (10), it is sufficient to compute the b-coefficient, and then find $a(\xi )$ using Eq. (11). If needed, we then can use both computed quantities to find the reflection coefficient (10). In practice, the b-coefficient is preferable, since when calculating the $r(\xi )$, in the case of a value of the $a(\xi )$ close to zero, the numerical error of the calculation greatly increases. We note that within the b-modulation concept, which has turned out to be the most efficacious NFDM method developed so far, we utilise the $b(\xi )$ functions as information carriers^24–27^.

### NF spectrum for the weakly-nonlinear case and threshold for soliton nucleation

Let us assume that the amplitude of our signal is small, say $|q(t)| \sim \varepsilon$, with $\varepsilon \ll 1$. Then, we can derive the following expansions for the NF scattering coefficients^14^:

12 $$\begin{aligned} a(\xi ) = 1 - \intop _{-\infty }^{\infty } dt_1 \intop _{-\infty }^{t_1} dt_2 \, e^{2 i \xi (t_1-t_2)} q(t_1) {{\bar{q}}}(t_2), \end{aligned}$$

up to $\varepsilon ^2$ (the next expansion term $\sim \varepsilon ^4$), and

13 $$\begin{aligned} b(\xi ) = -\intop _{-\infty }^{\infty } dt_1 \, e^{- 2 i \xi t_1} {{\bar{q}}}(t_1) + \intop _{-\infty }^{t} dt_1 \intop _{-\infty }^{t_1} dt_2 \intop _{0}^{t_2} dt_3 \, e^{2 i \xi (t_2-t_1-t_3)} {{\bar{q}}}(t_1) q(t_2) {{\bar{q}}}(t_3), \end{aligned}$$

up to $\varepsilon ^3$ (the next expansion term $\sim \varepsilon ^5$). With the accuracy up to $\varepsilon ^4$, we have for the reflection coefficient:

14 $$\begin{aligned} r(\xi ) = - \intop _{-\infty }^{\infty } dt_1 \, e^{- 2 i \xi t_1} {{\bar{q}}}(t_1) - \intop _{-\infty }^{\infty } dt_1 \intop _{t_1}^{\infty } dt_2 \intop _{-\infty }^{t_2} dt_3 \, e^{2 i \xi (t_2-t_1-t_3)} {{\bar{q}}}(t_1) q(t_2) {{\bar{q}}}(t_3) . \end{aligned}$$

So we see that the first linear term in $r(\xi )$ expansion is simply the conjugated FT of our signal up to the frequency scaling factor. Then, $r(\xi )$ from Eq. (14) differs from the expression for $b(\xi )$, Eq. (13), only by the terms $\sim \varepsilon ^3$ and higher, but the structure of both expressions is the same, and so the NFT-Net with the same structure can successfully recover both $r(\xi )$ and $b(\xi )$ if we explicitly train it for the recognition of the corresponding quantity. We believe that this also holds for any level of nonlinearity, maybe aside from the case when we are close to the soliton creation threshold and $r(\xi )$ displays sharp peaks^14^, Fig. 2]. But, in such a special scenario, it looks more efficient to use the NN to recover $a(\xi )$ and $b(\xi )$ profiles, as these do not typically display any singular behaviour.

Turning to the question of soliton appearance from a localised profile, the rigorous criterion for our having no embedded solitons can be formulated for single-lobe profiles as^73^:

15 $$\begin{aligned} \intop _{-\infty }^{\infty } |q(t)| \, dt < \pi /2, \end{aligned}$$

and the deterministic profiles used in our work have a much higher normalised energy. For more involved multi-lobe profiles, the soliton-creation threshold is typically higher, but we still had some profiles that contained solitary components, so we had to eliminate them. When we add noise to our signal that initially contains no solitons, a random modulation typically diminishes the probability of solitons appearance^74,75^. However, we checked out that all randomly perturbed signals used in our study did not contain a solitonic component as well.

**Figure 6:**
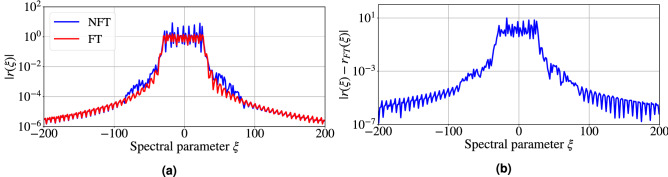
**(a)** Example of the amplitudes of Fourier spectrum (FT) and continuous nonlinear Fourier spectrum (NFT) for one of the training signals. **(b)** Example of the absolute value of the difference between Fourier spectrum and continuous nonlinear Fourier spectrum for one of the training signal. For both graphs signal energy $E_{{{\text {signal}}}} = 39.0$ in non-dimensional units.

To demonstrate the difference between the continuous NFT spectrum and the linear FT spectrum, we calculated (taking into account the necessary transformations and frequency scaling) both spectra for an example signal of the type used in our analysis. As the measure showing the distinction between the conventional Fourier and NF spectra, we use the norm of the difference: $|r(\xi ) - r_{FT}(\xi )|$, where $r_{FT}(\xi )$ is given by the first (linear) term in the expansion of $r(\xi )$, Eq. (14). Figure 6a shows an example of a nonlinear and conventional Fourier spectrum. The dependence of the difference on the spectral parameter $\xi$ for a typical signal from our testing set is shown in Fig. 6b. The critical decrease of the difference at $\xi$ region below $-100$ and above 100 occurs because the amplitude of the continuous spectrum at that region also tends to zero. The average maximal difference parameter value over the entire spectrum for all signals from the test dataset is $\approx 9$. This fact allows us to argue that the nonlinear effects are essential for the selected testing signals, despite their containing no solitons. Thus, the accuracy of the NFT-Net allows us to perceive the truly nonlinear effects.

### Numerical NFT computation

In our work we used the conventional forward NFT numerical method to generate training and testing data set pairs: the signal and its respective NF spectrum. For the computation of continuous NF spectrum associated with a given profile *q*(*t*) (containing no solitons) having the form of Eq. (2), we used the exponential scheme ES4 from the FNFT package^58^ (non-fast realisation). It has the accuracy proportional to the fourth power of the time sample size, $\sim (\Delta t)^4$. We note that there exists the fast realisation of the NFT processing with $\sim (\Delta t)^4$ accuracy^76^, which can potentially be used for efficient NFT-Net training.

### Complexity analysis

One of the important metrics in the development of signal processing tools is the complexity of the processing device, i.e. the number of elementary arithmetic operations that the processing unit employs to reach its goal. Quite often we need to analyse the interplay between the complexity and accuracy of the processing unit. Thus, here we perform the complexity analysis for the NFT-Net.

In our case, we concentrate only on the number of multiplications, since in practical implementation the computational complexity of addition operations is negligible. The number of real multiplications needed for the forward propagation of the model, as introduced in^77^ for several types of NN layers, is also used to calculate the computational complexity of the NFT-Net in this paper.

The overall complexity *C* of the NFT-Net can be presented as the sum of two constituents: the complexity of densely-connected block $C_{{{\text {dense}}}}$ and the complexity of convolutional block $C_{{{\text {conv}}}}$. For the calculation of $C_{{{\text {dense}}}}$ the same formula as in^77^ can be used, where we have $n_i$ inputs, $n_1$ neurons in the hidden layers, and $n_o$ outputs, and the complexity is defined as:

16 $$\begin{aligned} C_{{{\text {dense}}}}= n_1*(n_{i} + n_{o}) {,} \end{aligned}$$

In the case of the convolution layer, we can change the equation given in^77^ to measure the generalised convolutional layer complexity by taking into account the number of filters *f* and kernel size *k*, as well as the effect of padding *p*, stride *s*, and dilation *d*. The complexity $C_{{{\text {conv, layer}}}}$ for one layer when the input shape is [$L_{in},Q_{in}$], is specified as follows:

17 $$\begin{aligned} C_{{{\text {conv, layer}}}} = k* Q_{in} * f *\left( \frac{L_{in} + 2*p -d*(k-1)-1}{s} +1\right) {,} \end{aligned}$$

where $Q_{in}$ denotes a number of channels, $L_{in}$ is a length of signal samples sequence. Therefore, the total complexity of the NFT-Net used in this paper in terms of real multiplications per output sequence (1024 complex valued points) is:

18 $$\begin{aligned} C = 2*(C_{{{\text {conv, 1}}}}+C_{{{\text {conv, 2}}}}+C_{{{\text {conv, 3}}}}+C_{{{\text {dense}}}}) {,} \end{aligned}$$

where the factor 2 in front appears due to the use of two identical NNs to predict the real and imaginary parts of the continuous NF spectrum. Turning to our optimised architecture, to process 1024 complex signal samples, the following number of multiplication operations for the optimised architecture is required:

19 $$\begin{aligned} C = 2*[10*2*10*1006+ 18* 10*15 * 972 + 14*15*10*320 + 4096*(3200+1024)] = 41598208 {.} \end{aligned}$$

For comparison, processing a signal consisting of 1024 points using FNFT methods from Ref.^78^ requires 3885572 FLOPs (note that this is not the number of multiplications, so the direct comparison with the number from Eq. (19) is somewhat difficult). Generally, for the computation of *N* points in the NF spectrum from *N* point in *t*-domain, the non-fast NFT methods^72^ typically require $N^2$ FLOPs, while the fast methods need $N \log ^2N$ FLOPs^58,78^. From this perspective, the complexity of the current NFT-Net corresponds to that of non-fast NFT methods. However, some techniques can be further used to reduce the NN’s complexity^79^.
